# Effects of Replacing Grass with Foliage on Growth Rate and Feed Intake in Goats—A Systematic Review and Meta-Analysis

**DOI:** 10.3390/ani11113163

**Published:** 2021-11-05

**Authors:** Cecilia Kronqvist, Daovy Kongmanila, Ewa Wredle

**Affiliations:** 1 Department of Animal Nutrition and Management, Swedish University of Agricultural Sciences, 75007 Uppsala, Sweden; Ewa.Wredle@slu.se; 2 Faculty of Agriculture, National University of Laos, Vientiane P.O. Box 7322, Laos; d.kongmanila@nuol.edu.la

**Keywords:** dry matter intake, average daily gain, foliage inclusion level

## Abstract

**Simple Summary:**

Goats are important as food sources and livelihood in several areas of the world, mainly in low-income countries. Free-range browsing is common, but to increase productivity and improve health, supplemental or complete feeding is increasing. Ruminant feeding has often been using grasses, but as goats are browsers, they may benefit from including foliage in the diet. This systematic review and meta-analysis focuses on the effect of including foliage in goat diets. The results show that foliage was often more nutrient-rich and increased dry matter intake as well as average daily weight gain in goats.

**Abstract:**

Small ruminants such as goats have a higher preference for browse species than cattle and sheep. In a meta-analysis of 42 papers describing 117 experimental treatments found by a search performed in June 2021 in PubMed and Web of Knowledge, we examined the general effect of including foliage in the diet of goats, replacing grasses, on dry matter intake and average daily weight gain. The inclusion requirement for a paper was that it described a controlled trial with a control diet of grass and with grass replaced by foliage in the experimental diet. Publication bias was estimated by calculating the Fail-safe n. Random effects analyses were conducted, using effect size calculated as Hedges’ d. The results showed that inclusion of foliage increased feed intake (Hedges’ d = 1.350, SE = 0.388) and average daily weight gain (Hedges’ d = 1.417, SE = 0.444) compared with a grass-based control. The positive effect of foliage inclusion on dry matter intake was associated with lower neutral detergent fiber (NDF) and higher crude protein (CP) in the foliage than in the grass it replaced. The positive effect on average daily weight gain was associated with higher CP concentration in the foliage than in grass. Foliage inclusion level showed a quadratic relationship with dry matter intake, with maximum dry matter intake achieved at a level of 50–60%. There was wide variation between the studies reviewed, and this variation was not reduced by subgroup analysis based on different kinds of foliage. In conclusion, the addition of foliage to goat diets can increase feed intake and daily weight gain, as an effect of the dietary preferences of goats and of generally higher nutritional value in foliage species compared with natural/semi-natural grass species.

## 1. Introduction

There are more than a billion domestic goats worldwide, distributed over almost all continents [1]. In most parts of the world, goat production is typically less intensive than cattle or sheep production. Most goats are kept by smallholder farmers in low-income countries or in developing countries, especially in Asia and Africa. In areas with limited possibilities for food production, goats can strongly contribute to human food security and will likely become even more important in light of climate change and environmental changes. However, their contribution to the household food supply is not always evident in official records [2]. Goats, for milk and meat production, are often reared in extensive free-range systems, especially on smallholder farms in Asia and Africa [3]. Their diet in such systems is at least partly determined by natural or semi-natural vegetation that they locate and select themselves, and growth rate and milk production in these low-input systems are typically quite low [3]. To intensify production and animal health, increased use of housing and better feeding is necessary. Increased demand for goat products and decreased land availability for free-range browsing can be future drivers for intensification of goat production.

Since goats are browsers, given the choice, they will include a higher proportion of foliage in their diet than sheep and cattle [4]. However, goats can also thrive and grow on a grass-based diet [5]. If the production, harvest and storage system for grasses is well-developed, as in many industrialized countries, conserved grass is often used as the basal diet for goats in intensive production systems [6]. However, studies have tested the effect on feed intake and growth in goats of replacing all or parts of the grass in a grass-based diet with foliage of different kinds. This foliage can originate from the production of food crops, such as cassava (*Manihot esculenta*), or can be grown as feeds, such as leucaena (*Leucaena spp*.) [7,8,9]. Some foliage species have a high crude protein (CP) concentration and a low fiber concentration, but also often contain high levels of anti-nutritional factors that may have negative effects on feed intake and growth [10].

The aim of this review was to evaluate the overall effect of replacing grass with foliage in the diet of goats, focusing on feed intake and growth performance. Possible associations between observed effects and the nutritive value of the foliage and grass were assessed.

## 2. Materials and Methods

### 2.1. Search Process and Selection of Papers

This review used a systematic approach mainly according to the guidelines of the Preferred Reporting Items for Systematic Reviews and Meta-Analyses (PRISMA) [11], but did not establish a protocol, and the review was not registered.

**Figure 1:**
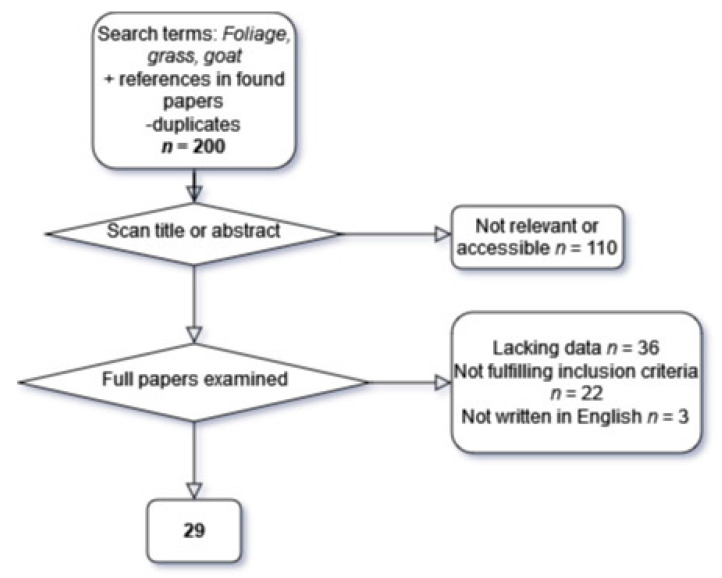
Flowchart for selection of publications for the meta-analysis.

A systematic and thorough literature search was conducted during January 2021, using the databases Web of Knowledge and PubMed. Additional papers were found in the reference list of some of the papers located by the search. The selection flowchart is presented in Figure 1. After duplicates were removed, a brief scan of the title or abstract was conducted, removing papers that were not relevant or because of wrong species, irrelevant layout of the study or, in one case, non-accessible full-text versions. Full texts were retrieved to be investigated more carefully. One reviewer screened all papers and extracted the data. This may have caused bias as retrieval of papers and data extraction is a process that is subjective to some extent.

The inclusion criterion for a study was that it included a control group fed grass and an intervention group fed foliage. All kinds of foliage and grasses were accepted as treatment and control, respectively. Furthermore, no restrictions were set regarding the level of grass or foliage inclusion in the diet, or on the complementary feed ingredients used in addition to grass or foliage. All grass conservation methods and all feeding methods for the grass and foliage parts of the diet were accepted. However, studies in which foliage was added to the diet but did not replace all or parts of the grass fraction in the diet were excluded. For practical reasons, papers where data were found only in sections written in languages other than English were not included.

### 2.2. Data Extraction

No protocol was prepared, and the review was not registered. Data, including foliage species, age of goats, inclusion level of foliage, sample size, experiment duration, dry matter intake (DMI, mean and SD) and average daily weight gain (ADG, mean and SD), from each experimental treatment were extracted and imported into Excel. If standard deviation (SD) for dry matter intake or average daily weight gain was not provided in a paper, an estimated SD was calculated as standard error (SE) of the mean times the square root of the number of replicates (n) in control and treatment groups. This calculation was required for most papers included. Many of the papers stated only one SE for all treatments combined, but then applied that value to individual treatment means. The commonly occurring case of several different treatments associated with the same control group within an experiment was handled according to [12]. This meant that when the difference between treatments within an experiment (e.g., amount or type of foliage) was actually analyzed in the model, they were kept as separate data lines. When the difference was not analyzed in the model, all treatments were combined into a mean, weighted by the number of replicates in each treatment (with a pooled SD) and then compared with the control treatment.

Percentage of foliage in the diet was calculated based on actual intake (dry matter (DM) basis), while average daily weight gain was calculated by dividing average weight gain during an experimental period (in some cases calculated from body weight before and after the experiment) by the number of days in that experimental period. Not all papers in the dataset reported enough data to extract all the required variables. In total, 107 experimental treatments in 42 different experiments were included in the analysis. Of these, 30 experiments were suitable for use to estimate the effect of foliage on dry matter intake, and 22 experiments were suitable for use to estimate the effect of foliage on average daily weight gain.

### 2.3. Statistical Analysis

The meta-analysis was performed using the free software program OpenMee [13], which is based on the “metafor” package in R (R Foundation). The effect size in each comparison between treatment and control was calculated as Hedges’ d [14], both for daily growth and for daily dry matter intake. Hedges’ d is a corrected, less biased version of Hedges’ g, which in turn is better adapted to small sample sizes than Cohen’s d [14]. Separate random effects’ analyses were performed to examine the overall effect of foliage compared with grass, where one observation per control group was included. Subgroup analyses were also performed, where the experimental treatments were divided into different foliage types, with one observation per foliage type used in each experiment. Finally, meta-regression was carried out, with the level of foliage inclusion in the diet as a continuous variable and with one observation per level of foliage used in the experiments. Experimental treatments that reported CP and neutral detergent fiber (NDF) concentration in foliage and control treatments were used to estimate the effect of CP and NDF concentration in foliage and grass on dry matter intake and average daily weight gain. As there was evidence of covariation, manifested as a negative relationship between NDF and CP concentration in foliage, separate analyses were performed for these two nutrients to avoid confounded results. The significance level was set at 0.05. Heterogeneity was assessed by judging the I^2^ value [15], a statistic that shows the proportion of variation between studies that cannot be assigned to random sampling variation. A common interpretation of the I^2^ value is that a value below 25%, 50% and 75% represents low, medium and high variation between studies, respectively.

Publication bias was estimated by calculating Fail-Safe n according to the Rosenberg method [16]. This method estimates the number of experiments resulting in non-significant differences that could be added to the statistical analysis before the result of the meta-analysis becomes non-significant. It is calculated based on the assumption that studies with non-significant results have a lower probability of being published in an accessible way than studies with significant results. To further evaluate the risk of publication bias, effect sizes and SD in the studies included in the review were plotted in a funnel plot, to visually evaluate the potential risk of biased under-retrieval of studies with small sample sizes and small or non-significant effects.

## 3. Results

The experimental groups in the studies included in the review contained between 3 and 16 goats. When groups were combined to take the mutual controls into account in assessing the overall effect of foliage inclusion, the maximum number of observations in the data points included in the statistical analysis increased to 48.

The characteristics of the experiments and the experimental diets in the papers included in the review are shown in Table 1. The nutritional value of the foliage used in experiments included in the analysis was higher than that of the control grasses, with an average CP content of 184 (range 47–302) g/kg DM in the foliage, compared with an average of 97 (range 31–189) g/kg DM for the grasses used in the control diets. For NDF, the foliage contained 178–749 g/kg DM, while the grasses used as controls contained 275–850 g/kg DM. The inclusion level of foliage ranged between 11% and 100% of DM. Goat breeds and foliage species included in the experiments are listed in Table 2.

Foliage in the diet was associated with increased DMI (Hedges’ d value: 1.350, SE: 0.388, *p* < 0.001). It was also associated with increased ADG (Hedges’ d value: 1.417, SE: 0.444, *p* < 0.001). Upon including the percentage of foliage in the diet as a continuous variable, the slope was not significant for either DMI or ADG, but for DMI, there was a significant quadratic relationship with percentage of foliage in the diet, with the resulting prediction Equation (1) being:

Hedges’ d for DMI = −2.166 + (0.217 × % foliage) − (0.002 × (% foliage)^2^) (1)

This implies that the maximum positive effect of foliage inclusion occurs at an inclusion rate of between 50% and 60% of foliage in the diet, and that inclusion of 100% foliage in the diet will not provide any beneficial effect on DMI compared with a grass-based diet. The average level of foliage inclusion in experiments included in the analysis was 42%, and most experiments included between 20% and 80% of foliage in the treatments.

The effect of foliage inclusion on DMI was positively affected by the low nutritive value of the grass that was replaced by foliage, i.e., increased NDF concentration in the diet (0.16 increase in Hedges’ d per 1% increase in NDF concentration compared with grass, *p* = 0.005), and by the decreased CP concentration in the grass (0.48 increase in Hedges’ d per 1% decrease in CP concentration compared with grass, *p* = 0.008). The effect of foliage inclusion on ADG was positively affected by the CP concentration in foliage (0.18 increase in Hedges’ d per 1% increase in foliage CP concentration, *p* = 0.012) and by the decreasing CP concentration in the grass it replaced (0.3 increase in Hedges’ d per 1% decrease in grass CP concentration, *p* = 0.002).

In all analyses, the variation between studies, as measured by the I^2^ value, was high (I^2^ value > 0.75 in all cases). This was not substantially reduced by subgroup analysis within foliage type, although the heterogeneity within a few subgroups was markedly decreased (data not shown). Several of the classified foliage types were only represented by one single study or by one group of researchers, while some foliage types were examined in multiple studies by different authors.

**Figure 2:**
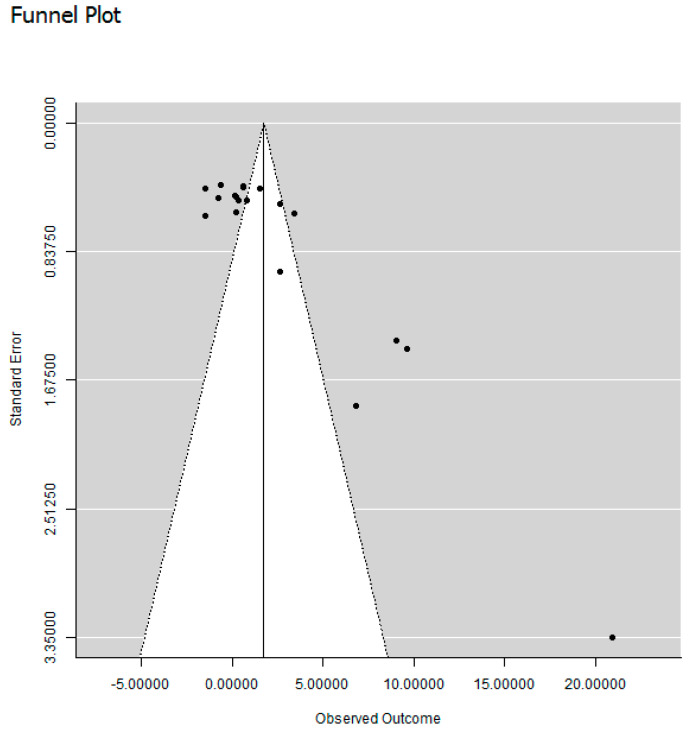
Funnel plot illustrating the risk for publication bias for the overall analysis of the effect of foliage inclusion on dry matter intake. Observed outcome is measured as Hedges’ d.

Visual inspection of the funnel plot for the studies included in the review revealed a great risk of publication bias, with larger effect size estimates in smaller studies (Figure 2). However, Rosenberg’s Fail-Safe n, estimating the number of studies with a non-significant effect of foliage inclusion that could be added to the analysis before the overall result became non-significant, was 296 for the effect of foliage on dry matter intake and 131 for the effect of foliage on ADG, both of which can be considered high.

## 4. Discussion

This meta-analysis of 42 papers describing 117 experimental treatments revealed that the inclusion of foliage in the diet of goats, as a substitute for natural/semi-natural grasses, increased DMI and improved ADG. Goats, being small ruminants, are at a disadvantage compared with cattle in terms of the ability to utilize low-quality feed, e.g., rough grasses, as they have a higher maintenance energy requirement per kg body weight but lower feed intake capacity than cattle [43,44]. Free-ranging goats resolve this by selecting more nutritious foliage over grass species. In the present meta-analysis, the inclusion of foliage in the diet had a large effect on both DMI and ADG, with estimated Hedges’ d values well above 1 in both cases, indicating that the study results are robust. The results of regression analysis showed that the effect size increased with increased nutritional quality of the foliage used in the treatments and decreased with increased nutritional quality of the grasses in the control diet. This indicates that this was at least partly a result of generally higher CP concentration and lower fiber concentration in the foliage compared with the grasses in the experimental diets. It has been shown previously that DMI in goats is negatively affected by the NDF concentration, and positively affected by the CP concentration, of the plants in their diet [45]. A previous meta-analysis [46] found positive effects on DMI and ADG from the inclusion of tree foliage in the diet of goats, although sample size was not included in the statistical analysis.

There are several reasons why foliage in the diet could lead to increased intake and growth when replacing grass. The higher fiber concentration can be suspected to increase time spent eating and ruminating per kg of DM [47]. Additionally, the variation caused by replacing parts of the forage in the diet may increase feed intake, as goats given choices have been shown to vary their intake among species [48]. Condensed tannins, which are more common in foliage compared to grasses, may increase DMI in goats when fed at a moderate level, although the effect on ADG is limited [49]. Tannin-containing plants can also affect rumen microbe composition.

In many of the experimental treatments included in the analysis, foliage was fed freshly harvested. However, improved methods for preservation and storage of foliage and increased use of conserved foliage in the diet could be a way to improve goat production and facilitate intensification, including in smallholder farming. The feed value of conserved foliage can be as good as that of the fresh material. For example, the authors of [29] tested cassava foliage in fresh and ensiled form and found no differences in DMI or growth performance in goats between the treatments. They also found that the ensiled cassava had a higher CP concentration than the fresh material. However, it should be noted that their study was performed on goats infected by intestinal parasites and that the overall feed conversion ratio was relatively low [29].

The foliage species tested in the different experiments included in this meta-analysis differed depending on the region in which they were grown. The range of plants included comprised species exclusively grown for feed production, such as leucaena (e.g., used in [35]) and species commonly used for human food production, such as cassava [33]. In a survey in three districts in south-eastern Nigeria, the authors of [50] found that geographical variations resulted in somewhat different plant species being preferred in small ruminant nutrition. Their survey also showed that the choice of plants was at least partly decided by food production in the area, as several of the plant materials used were by-products of human food production. In addition, exotic plants that had been purposely introduced in the flora were commonly used in ruminant nutrition [50]. Use of foliage that is a by-product of food production, e.g., post-harvest residues or kitchen waste, may be a viable way to develop future goat production, transforming it from pure free-range browsing to more controlled feeding, whether in a drive for intensification or in response to decreasing availability of land for free-range goat herding [51]. The foliage of several food crops, as a by-product of food preparation or as harvest residues, is suitable for use as feed in goat production. Use of food crop residues in goat feeding also has the potential to improve environmental sustainability, as measured by, e.g., global warming potential, eutrophication potential and acidification [52], compared with the use of grasses produced solely for ruminant feeding purposes.

There was great variation between the experiments included in this meta-analysis, which was a result of differences between goat breeds, diets, environments and layout used in the studies. However, the overall results showed a positive effect on both DMI and ADG of replacing grasses with foliage in goat diets. The Fail-Safe n value indicated a large margin for adding studies with non-significant results, showing that the results are fairly robust, although publication bias can be expected. The wide range of plant materials, species and harvest times, both for foliage and grasses, together with different inclusion levels and varying use of concentrates, caused a wide variation in experimental diets. Goat feed intake has been shown to be affected not only by diet composition, but also by feed presentation or feed processing method, e.g., feeding the diet chopped or not [53], but the method of feeding the diet or the feed processing method was not considered in the present analysis. According to [54], much information can be gained from a meta-analysis by exploring causes of heterogeneity using different methods. In the present case, dividing the experiments included in the analysis into subgroups based on foliage type did not greatly decrease the heterogeneity in general, but reduced it for some subgroups. This reduction in heterogeneity could strengthen the conclusions for these subgroups, but may also be caused by some foliage types being studied more often in some research subgroups, due to geographical variations or differing interests. Alternatively, study design, animal material, diets, etc., may have been more similar, reducing the heterogeneity for those foliage types. In addition, several foliage types were only studied in one or two experiments, which could explain why subgrouping did not decrease the heterogeneity.

The amount of foliage included in the diet showed a quadratic relationship with the effect of foliage inclusion on DMI, resulting in the highest estimated improvement in total DMI when foliage was included at about half of the total dry matter intake. A quadratic response to foliage, resulting in an optimum inclusion level for foliage in the diet above which DMI decreases, has been reported previously [55]. In a study specifically on foliage from trees, the authors of [46] found 50% of maximum DMI to be the optimum level of foliage inclusion in the diet. This may be an effect of the commonly higher levels of anti-nutritional factors in foliage compared with grasses [56]. However, preference studies have shown a limited effect of the level of tannins on goat DMI when comparing tanniferous plants [57]. The amount of foliage in the diet did not have any significant effect on ADG. This was surprising, as there was an effect of foliage inclusion compared with no foliage, and effects of the nutrient concentrations in foliage and grass. This could be a result of the smaller number of trials that measured body weight and were therefore used to calculate ADG, resulting in lower statistical power.

Many of the studies included in the meta-analysis consisted of small experimental groups, with an overall average number of only five goats in each group. Nevertheless, most studies located and included in the review reported a significant difference between the treatments. The funnel plot of the studies showed that the effect size was much larger in studies with large standard deviation, which is often a result of small sample size. This indicates that the likelihood of finding a study with a small effect size, which may correspond to the likelihood of publication of such a study, increases with the increasing number of animals in the treatment groups. This may indicate in turn that studies with small sample sizes have been performed but are not published, since the results did not show a sufficiently high effect of foliage inclusion in the diet of goats. This was also evident from the asymmetry in the funnel plot. However, asymmetry in funnel plots has also been shown to depend on how the plot is constructed, and interpretation of the results shown in the plot could be altered by using other effect metrics [58]. Post-hoc power calculations showed that the average number of animals in the studies included in the present meta-analysis gave a low probability of identifying significant differences between treatments, which may indicate publication bias. To improve research on goat feeding, improved design of feed trials and larger numbers of animals are suggested.

## 5. Conclusions

This meta-analysis showed that inclusion of foliage in the diet of goats, as a replacement for grass, increases dry matter intake and average daily weight gain. At least part of this effect may stem from the improved nutritional content of diets including foliage with higher crude protein levels and lower levels of fiber (measured as NDF) than natural grasses. There was wide variation between the studies included in the analysis, but Fail-Safe n values indicated that the general findings on the effect of foliage supplementation on dry matter intake and daily weight gain are robust.

## Figures and Tables

**Table 1:** Selected characteristics of the 42 papers describing 117 experimental treatments included in the meta-analysis. Publication year between 1990 and 2016, median 2003.

Item	N	Mean	Median	Minimum	Max	SD
Experimental length (days)	42	69.3	69	7	365	60.7
Initial age of goats (days)	36	n.a	275	99	Adult	n.a
Amount of foliage (% of DM)	42	43.7	48	15	100	25.7
CP control (g/kg DM)	39	97	97	31	189	41
CP foliage (g/kg DM)	39	184	185	55	302	55
NDF control (g/kg DM)	29	650	704	275	850	162
NDF foliage (g/kg DM)	29	438	403	178	749	140
Initial goat BW (kg)	41	24	15.5	6.93	40	10.2
Control treatment DMI (g)	42	537	499	190	430	290
Foliage treatment DMI (g)	42	571	584	119	1290	214
Control treatment ADG (g)	30	26.6	50	−53	98	32.7
Foliage treatment ADG (g)	30	47.9	54	−84	152	29.8

N: Number of studies, SD: standard deviation, DM: dry matter, CP: crude protein, NDF: neutral detergent fiber, BW: body weight, DMI: dry matter intake, ADG: average daily weight gain.

**Table 2:** Description of studies.

Foliage	Year	N	Country	Breed	Reference
*Acacia senegal* *Pterocarpus lucens* *Guiera senegalensis*	2008	8	Burkina Faso	Sahel goats	[17]
*Atriplex nummularia*	2016	6	Egypt	Balady	[18]
*Moringa oleifera*	2016	16	Egypt	Anglo-Nubian	[19]
*Erythrina abyssinica*	1993	6	Ethiopia	Local	[20]
*Azadirachta indica* *Acacia Senegal*	2016	5	Etiopien	Short-eared Somali	[21]
*Acacia tortilis*	2005	4	Kenya	SEAG	[22]
*Gliricidia sepium*	1999	4	Kenya	Toggenburg × Saanen	[23]
*Leucaena leucocephala*	1991	4	Nigeria	West African dwarf	[24]
*Piliostigma thonningii* *Daniellia oliveri* *Afzelia africana* *Pterocarpus erinaceus* *Annona senegalensis*	2015	4	Nigeria	Red Sokoto	[25]
*Olea folium*	2016	8	Tunisia	Local	[26]
*Leucaena leucocephala* *Calliandra calothyrsus*	1992	6	Zambia	Local	[27]
*Manihot esculenta* *Flemingia macrophylla* *Desmanthus virgatum* *Musa*	2001	3	Cambodia	Local and Bach Tao	[28]
*Manihot esculenta*	2009	6	Cambodia	Local	[29]
*Leucaena leucocephala*	1998	8	India	Jamunapuri	[30]
*Leucaena leucocephala*	1998	9	India	Jamunapuri	[31]
*Erythrina variegata*	2012	6	Laos	Ma T’ou	[9]
*Stylosanthes guianensis*	2003	9	Laos	Local	[32]
*Manihot esculenta*	2007	8	Laos	Local	[33]
*Artocarpus heterophyllus* *Flemingia macrophylla*	2001	8	Vietnam	Bachthao Barbari × Bachthao Jamnapary Bachthao	[34]
*Manihot esculenta* *Artocarpus heterophyllus* *Leucaena leucocephala*	2003	6	Vietnam	Bachthao Bachthao × Barbari	[35]
*Robinia pseudoacacia* *Populus* spp.	1996	4	USA	Angora	[36]
*Quercus virginiana* *Juniperus ashei*	1999	10	USA	Angora	[37]
*Tipuana tipu* *Calliandra calothyrsus*	2000	3	Australia	Australian Cashmere	[38]
*Talfairia occidentialis*	2007	5	Samoa	Anglo-Nubian	[5]
*Erythrina variegata* *Gliricidia sepium* *Leucaena leucocephala*	2004	4	Samoa	Anglo-Nubian × Fiji	[39]
*Erythrina variegata* *Gliricidia sepium* *Leucaena leucocephala*	2004	4	Samoa	Anglo-Nubian × Fiji	[40]
*Sesbania grandiflora*	1992	4	Samoa	Fiji × New Zeeland	[41]
*Pithecellobium dulce* *Gliricidia sepium* *Haematoxylum brasiletto*	2013	5	Mexico	Creole goats	[42]

N = Number of goats in the control treatment group. The foliage treatment groups were the same size in almost all cases.

## Data Availability

The data presented in this study are available upon request.
